# Supplementary material for: In Vitro Studies of Endophytic Bacteria Isolated from Ginger (Zingiber officinale) as Potential Plant-Growth-Promoting and Biocontrol Agents against Botrytis cinerea and Colletotrichum acutatum
Source: Plants (Basel). 2023 Nov 30;12(23):4032. doi: 10.3390/plants12234032 (PMC10708395; doi:10.3390/plants12234032)
Supplement: Supplementary file 1 [file plants-12-04032-s001.zip › plants-2736048-supplementary.pdf]

SUPPLEMENTARY MATERIAL

Table S1. Percentage inhibition of growth in phytopathogenic fungi *B. cinerea* and *C. acutatum* subsequent to co-cultivation with ginger endophytic bacteria in LB medium after 7 days of incubation at 25°C.

**Isolates**	**Identification**	**Inhibition of *B. cinerea* B05.10 (%)**	**Inhibition of C. acutatum IMI34849 (%)**
J2	*Lelliottia amnigena*	96.73 ± 0.09	91.85 ± 0.45
J11		97.14 ± 0.44	90.13 ± 1.59
J12		97.40 ± 0.07	87.59 ± 0.31
J21		96.33 ± 1.38	73.81 ± 0.91
J29		97.17 ± 0.21	95.31 ± 0.49
J23	*Lysinibacillus capsici*	96.80 ± 0.17	91.10 ± 0.86
J24		96.13 ± 0.32	87.37 ± 0.60
J26		96.96 ± 0.55	90.50 ± 0.27
J22	*Lysinibacillus macroides*	97.25 ± 0.43	92.19 ± 0.17
J25		97.11 ± 0.38	92.82 ± 0.89
J15	*Kocuria polaris*	96.58 ± 0.29	75.05 ± 0.18
J17		95.98 ± 1.20	88.01 ± 1.59
J19		96.46 ± 0.50	90.21 ± 1.52
J16	*Agrococcus citreus*	97.08 ± 0.73	87.67 ± 0.97
J28		97.26 ± 0.56	91.28 ± 0.75
J14	*Agrobacterium tumefaciens*	96.86 ± 0.84	91.52 ± 1.04
J20	*Zymobacter palmae*	95.22 ± 0.80	83.17 ± 0.60
J30	*Acinetobacter schindleri*	94.13 ± 1.66	91.85 ± 0.45
J5	*Mycolicibacterium* sp.	96.60 ± 0.75	90.13 ± 1.59

Values are expressed as mean ± standard deviation of triplicate readings.

Table S2. Quantification of indole acetic acid (IAA, µg/mL) synthesized by the isolates after culturing each bacterial strain on King-B medium in the presence and absence of the amino acid tryptophan (Trp). Incubation occurred for 48 hours at 25°C with continuous shaking.

Isolate	Identification	[IAA] with Trp	[IAA] without Trp
J2	*Lelliottia amnigena*	4.02 ± 0.09	2.51 ± 0.06
J11		1.39 ± 0.14	0.17 ± 0.06
J12		3.09 ± 0.12	0.17 ± 0.06
J21		2.33 ± 0.18	0.81 ± 0.06
J29		4.60 ± 0.20	0.00 ± 0.00
J23	*Lysinibacillus capsici*	4.56 ± 0.22	3.13 ± 0.14
J24		3.98 ± 0.35	1.24 ± 0.16
J26		2.22 ± 0.13	2.93 ± 0.29
J22	*Lysinibacillus macroides*	0.77 ± 0.06	0.13 ± 0.02
J25		3.42 ± 0.08	2.51 ± 0.04
J15	*Kocuria polaris*	2.31 ± 0.17	0.00 ± 0.00
J17		2.90 ± 0.14	1.47 ± 0.09
J19		1.49 ± 0.14	0.61 ± 0.09
J16	*Agrococcus citreus*	4.54 ± 0.31	4.54 ± 0.31
J28		0.81 ± 0.09	0.81 ± 0.09
J14	*Agrobacterium tumefaciens*	2.09 ± 0.08	2.09 ± 0.08
J20	*Zymobacter palmae*	1.26 ± 0.10	1.26 ± 0.10
J30	*Acinetobacter schindleri*	0.07 ± 0.01	0.00 ± 0.00
J5	*Mycolicibacterium* sp.	1.04 ± 0.06	1.04 ± 0.06

Values are expressed as mean ± standard deviation of triplicate readings.
